# Early Administration of Bosentan in High‐Risk COVID‐19 Outpatients at Risk of Sarcopenia: A Randomized, Double‐Blind, Placebo‐Controlled Trial

**DOI:** 10.1002/jcsm.13753

**Published:** 2025-03-04

**Authors:** Shaahin Shahbazi, Erfan Shahbazi, Farid Zayeri, Zahra Vahdat Shariatpanahi

**Affiliations:** ^1^ Department of Internal Medicine, Faculty of Medicine Ilam University of Medical Sciences Ilam Iran; ^2^ School of Medicine Tehran University of Medical Sciences Tehran Iran; ^3^ Department of Biostatistics, Faculty of Paramedical Sciences Shahid Beheshti University of Medical Sciences Tehran Iran; ^4^ Department of Clinical Nutrition and Dietetics, Faculty of Nutrition and Food Technology, National Nutrition and Food Technology Research Institute Shahid Beheshti University of Medical Sciences Tehran Iran

**Keywords:** Body Composition Analyzer, endothelin, long COVID, muscle performance, muscle strength

## Abstract

**Background:**

Endothelial damage induces myofibrillar breakdown and muscle degradation in COVID‐19 infection. There is a relationship between increased endothelin‐1 synthesis and sarcopenia. We evaluated the preventive effect of early bosentan therapy as an endothelin receptor blocker in sarcopenia in high‐risk outpatients with COVID‐19 infection.

**Methods:**

From 15 December 2021 to 15 August 2023, patients within 3 days of the onset of signs and symptoms were randomly assigned to receive bosentan, 62.5 mg, or placebo, twice daily from enrollment for 30 days. The primary outcome was disease progression (death or hospitalization within 15 days after randomization), and the data for this outcome have been previously published. Sarcopenia as a secondary outcome was assessed prospectively at 3, 6, 9 and 12 months after randomization using the criteria of the Asian Working Group for Sarcopenia (AWGS) 2019 (IRCT.ir, IRCT20211203053263N1).

**Results:**

A total of 313 patients (156 bosentan group, 157 controls) were included in the analyses, which were performed under the intent‐to‐treat principle. Overall, the incidence of sarcopenia was 8.6% (*n* = 27). Nineteen (73%) had severe sarcopenia. At the 3‐month follow‐up, the incidence of sarcopenia was 8.3% in the total population, with the significant risk difference (RD) of −10.17% in the bosentan group versus the control group. The incidence in the total population and RD in the bosentan group versus the control group at months 6, 9 and 12 were 8.6% (RD: −10.81%, *p* < 0.001), 8.3% (RD: −10.17%, *p* = 0.001) and 5.4% (RD: −6.99%, *p* = 0.003), respectively. During the study, 29 people developed severe COVID‐19 and were hospitalized. At follow‐up, sarcopenia occurred in four inpatients and 23 outpatients (*p* = 0.23). Mortality occurred in 5.1% (*n* = 16) of the total population, including 4 (1.3%) of the patients in the bosentan group and 12 (3.8%) of the patients in the placebo group (*p* = 0.069). None of the patients who died had sarcopenia. Bosentan did not cause any severe adverse events and was well tolerated.

**Conclusion:**

Early administration of bosentan may prevent sarcopenia in high‐risk outpatients with COVID‐19.

## 1 Introduction

Sarcopenia a disease of the skeletal muscles with loss of muscular mass and strength that is regarded as a common symptom of post‐COVID‐19 syndrome.

Post‐COVID syndrome or chronic COVID refers to a range of physical and psychological symptoms that develop during or after COVID‐19 and persist for more than 2 months. This syndrome has no alternative diagnoses [1].

Sarcopenic muscles undergo profound changes in their architecture, with both reduced fibre size and number, decreased number of satellite cells and accumulation of connective tissue and fat between the fibres [2]. In vivo studies have shown that endothelin‐1 (ET‐1) promotes fibrosis and senescence in cultured myoblasts in aging sarcopenia. Similar results have suggested a potential role for ET‐1 in the development of age‐related sarcopenia in mice [3]. ET‐1 was found to induce myoblast senescence and fibrosis through ETA receptor [3].

The hyper‐inflammatory state in COVID‐19 can activate and damage the endothelium with subsequent overexpression of vascular ET1, the most powerful vasopressor of endothelial cells that causes myofibril breakdown and muscle degradation due to mitochondrial dysfunction and autophagy [4, 5]. ET1 is also secreted from the endothelial lining of the airways, the cardiac cells, brain neurons, fibroblasts, macrophages and vascular smooth muscle cells [6].

Bosentan is a drug that blocks the endothelin receptors [7]. In this clinical trial with longitudinal outcomes, the full details of which are provided in the protocol, we hypothesized that the early administration of bosentan can prevent early and late outcomes of COVID‐19. Our published study has already reported the effectiveness of bosentan in reducing mortality and hospitalization in the acute stages of COVID [8]. The present report investigates the effect of bosentan on the occurrence of sarcopenia 3, 6, 9 and 12 months after the onset of the disease.

## 2 Materials and Methods

### 2.1 Study Design

This project is part of an ongoing randomized double‐blind, placebo‐controlled, phase 3 clinical trial with an add‐on design started on 15 December 2021 across two university hospitals in Ilam. It was registered in the Iranian Registry of Clinical Trials (IRCT20211203053263N1). The study protocol was approved by the appropriate institutional review boards and Ilam University of Medical Sciences Ethics Committee (IR.MEDILAM.REC.1400.164), and the work has been reported in line with Consolidated Standards of Reporting Trials (CONSORT) guidelines. Also, it was carried out in accordance with the Declaration of Helsinki of the World Medical Association. The results of the primary outcome of this study during the active infection have been published previously [8].

### 2.2 Trial Population and Randomization

We prospectively evaluated high‐risk COVID‐19 outpatients with at least one risk factor for severe COVID‐19 (age ≥50 years, hypertension, diabetes, serious heart conditions, chronic lung disease, chronic kidney disease, immunosuppression, sickle cell disease, BMI ≥ 25 and active cancer). The randomization and masking procedures are described in our previously published paper [8]. In summary, unvaccinated, laboratory‐confirmed COVID‐19 infected adults (≥18 years) were randomly assigned to receive either bosentan tablets, 62.5 mg, twice daily, or placebo, for 30 days, within 3 days of the onset of signs and symptoms. Patients with sarcopenia at baseline and patients who were permanently taking drugs affecting muscle function/mass were excluded (corticosteroids, statins, ACE inhibitors, sex hormones).

To evaluate the primary outcome of the study, which lasted 1 month from the onset of infection and the results of which have been published, vaccination was one of the exclusion criteria. After that, the patients received vaccination according to the protocol [9].

### 2.3 Sarcopenia Assessment and Diagnosis

Since there is no consensus definition of acute sarcopenia, the assessment and diagnosis of sarcopenia was made based on the Asian Working Group for Sarcopenia (AWGS) 2019 criteria, which is an age‐related sarcopenia [10]. Based on the AWGS criteria, sarcopenia is diagnosed with the loss of skeletal muscle mass accompanied by low muscle strength and/or physical performance. The presence of all three criteria corresponds to severe sarcopenia.

The patients were assessed for sarcopenia at baseline. They then had four more visits at 3, 6, 9 and 12 months after randomization.

Assessment was performed by measuring muscle strength and muscle performance.

Muscle strength was evaluated by the handgrip strength test. Hand grip strength was measured by a Jamar hydraulic dynamometer (Japan) while the participant was seated with 90° elbow flexion. The contraction of the dominant hand was measured three times with a 30‐s rest in between the measurements. The maximum value was taken for muscle strength. The cut‐off point for reduced muscle strength is taken as < 18 kg for women and < 28 kg for men aged ≥ 65 years. As we evaluated adults more than 18 years old, we used age‐gender specific thresholds for Iranians [11] to identify patients with low levels of muscle strength (Table S1).

Muscle performance was evaluated by measuring the time taken to walk 6 m at a normal pace from a moving start, without deceleration, and taking the average result of at least two trials as the recorded speed. The cut‐off point for reduced muscle performance was taken as < 1.0 m/s.

If impairment was found for either muscle strength or muscle performance, the patient was assessed for appendicular skeletal muscle (ASM) mass measurement for the diagnosis of sarcopenia.

ASM mass was measured by bioelectrical impedance analysis using Body Composition Analyzer Mediana, model i30 (Korea). The patients were instructed not to consume large amounts of water and not to exert sustained efforts 2–3 h before the measurements. To calculate appendicular skeletal muscle mass index (ASMI), the ASM mass in kilograms was divided by the square of body height in meters (ASMI = ASM/height^2^). ASMI < 7 kg/m^2^ for men and ASMI < 5.4 kg/m^2^ for women were taken as the cut‐offs to diagnose low muscle mass as described by AWGS 2019 criteria.

### 2.4 Nutritional Intake Assessment

Nutrition training was given to the patients in each visit based on the healthy eating plate.

Protein intake was planned as 1.2–1.5 g/kg in both study groups, calculated by a nutritionist, and was individualized according to the physiological characteristics and comorbidities of each patient. The patients were asked to keep a food record every month, which was then assessed by a nutritionist. The patients were also advised to walk for 20 to 30 min daily and were additionally given pearls with 50 000 international units of oral vitamin D supplementation to be taken once a month. They were also given a phone number to call whenever they had a question.

### 2.5 Outcome and Endpoints

The outcome was the occurrence of sarcopenia, with endpoints being 3, 6, 9 and 12 months after randomization. Data were collected by the researchers and analysed by a statistician working at the university and then interpreted by the authors. The authors guarantee the accuracy and completeness of the data and have reviewed and approved the final manuscript.

### 2.6 Statistical Analysis

This study is an ongoing clinical trial where the sample size was calculated based on primary outcome of the study, which was disease progression (death or hospitalization within 15 days after randomization). All the hypothesis tests were two‐tailed, with *p* < 0.05 denoting the level of statistical significance.

The chi‐square or Fisher exact tests were used to evaluate differences in the distribution of the categorical variables, and the Student t‐test was used to check differences in the distribution of the continuous variables between the intervention and placebo groups at baseline.

For the efficacy endpoints of sarcopenia, which had a binary response, the risk difference and relative risk for the bosentan group versus the placebo group as a reference were calculated.

A subgroup analysis of sarcopenia incidence with 95% confidence intervals was performed to assess whether the effect of bosentan differed by risk factors (age ≥ 50 years, hypertension, diabetes, smoking, serious heart conditions, chronic lung disease and BMI ≥ 25).

To make the sarcopenic data an intention to treat analysis, the missing observations were imputed as non‐sarcopenic, so the denominator matched the total number of randomized patients.

## 3 Results

**FIGURE 1:**
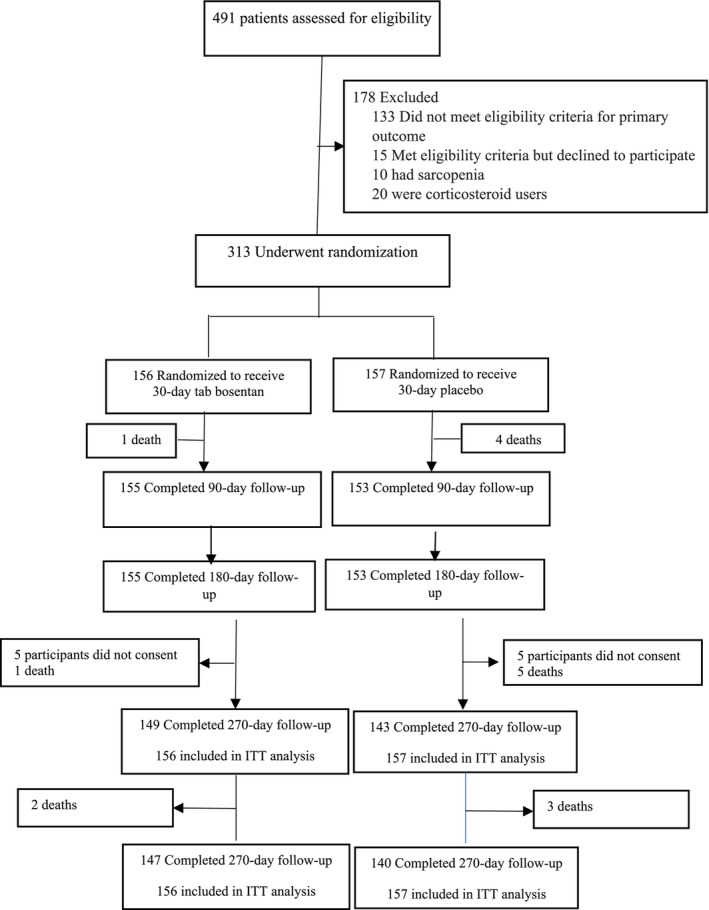
Flow chart depicting the study design.

From 15 December 2021 to 15 August 2023, a total of 313 patients (156 bosentan group, 157 controls) were prospectively evaluated for sarcopenia at 3, 6, 9 and 12 months, after randomization. Figure 1 presents the flowchart of the participants. The follow‐up visits were made in the corresponding months, with a deviation of 1 week. For the follow‐up visits at months 9 and 12, despite the phone calls, 10 participants (five in each group) did not cooperate with the researchers. Additionally, six participants died between the sixth‐ and ninth‐month visits, and five deaths occurred between the ninth‐ and 12th‐month follow‐up visits.

During the 12‐month follow‐up, the incidence of sarcopenia was 8.6% (*n* = 27). Nineteen (73%) had severe sarcopenia, four in the bosentan group and 15 in the control group. In the bosentan group, one patient had moderate sarcopenia, characterized by low muscle strength with decreased muscle mass. In the control group, seven patients had moderate sarcopenia, characterized by low muscle strength with decreased muscle mass in three subjects and low physical performance with decreased muscle mass in four patients.

Hospitalization during intervention occurred in 9.3% (29) of the total population, including four (1.3%) of the patients in the bosentan group and 25 (8%) of the patients in the placebo group (*p* < 0.001). At follow‐up, sarcopenia occurred in four inpatients and 23 outpatients (*p* = 0.23).

Mortality during 1‐year follow‐up was occurred in 5.1% (*n* = 16) of the total population, including four (1.3%) of the patients in the bosentan group and 12 (3.8%) of the patients in the placebo group (*p* = 0.069) (Table 1). None of the patients who died had sarcopenia.

**TABLE 1:** Demographic and clinical characteristics of patients.

Variable	Placebo (*N* = 157)	Bosentan (*N* = 156)	Total (*N* = 313)	*p*
Age, year	46·27 ± 13.18	45·69 ± 11.47	45.98 ± 12.34	0.67
Male	89 (56.7)	92 (59)	181 (57.8)	
Risk factors				
Age ≥ 50 years	67 (42.7)	60 (38.5)	127 (40.6)	0.45
BMI ≥ 25	80 (51)	76 (48.7)	156 (49.8)	0.69
Diabetes mellitus	96 (61. 1)	97 (62.2)	193 (61.7)	0.85
Hypertension	67 (42.7)	48 (30.8)	115 (36.7)	0.03
Tobacco or nicotine user	24 (15.3)	27 (17.3)	51 (16·3)	0.63
Serious heart condition	7 (4.5)	10 (6·4)	17 (5·4)	0.46
COPD	31 (19.7)	17 (10.9)	48 (15.3)	0.03
Number of risk factors				
1	33 (21)	42 (26,9)	75 (24)	0.22
2	59 (37.6)	61 (39. 1)	120 (38.3)	0.78
3	43 (27.4)	43 (27.6)	86 (27.5)	0.99
4	20 (12.7)	8 (5. 1)	28 (8.9)	0.01
5	2 (12.7)	2 (12.8)	4 (12.7)	0.99
Weight	76.98 ± 13.02	76.86 ± 13.66	76.92 ± 13.32	0.84
BMI (kg/m^2^)	25.05 ± 4.38	24.98 ± 4.13	25.01 ± 4.25	0.93
Hospitalization	25 (8)	4 (1.3)	29 (9.3)	< 0.001
Mortality	12 (3.8)	4 (1.3)	16 (5. 1)	0.069

*Note:* Data are reported as *X* ^2^ test (*n*, %) or t‐test (mean, SD).

Abbreviations: BMI, body mass index; COPD, chronic obstructive pulmonary disease.

Bosentan did not cause any severe adverse events and was well tolerated, and its consumption was stopped in only two patients because of a sharp increase in serum levels of aminotransferase.

Table 1 presents the baseline characteristic of the patients.

### 3.1 Sarcopenia

Table 2 shows the risk difference and relative risk of sarcopenia occurrence in the bosentan group versus the placebo group.

**TABLE 2:** The incidence rate of sarcopenia based on intention‐to‐treat and per protocol analysis.

Sarcopenia	Placebo, *n*/*N* (%)	Bosentan, *n*/*N* (%)	Risk difference, bosentan vs placebo (95% CI)	Relative risk, bosentan vs placebo (95% CI)	*p*
Within 3 months					
ITT	21/157 (13·4)	5/156 (3·2)	‐10.17% (−16.17 to −4.17)	0.24 (0.09 to 0.62)	0.001
PPA	21/153 (13·7)	5/155(3·2)	−10.5% (−16.62 to −4.37)	0.23	< 0.001
Within 6 months					
ITT	22/157 (14)	5/156 (3·2)	−10.81% (−16.9 to ‐ 4.71)	0.23 (0.09 to 0.60)	< 0.001
PPA	22/153 (14·38)	5/155(3·2)	−11.15% (−17.37 to −4.93)	0.22	< 0.001
Within 9 months					
ITT	21/157 (13·4)	5/156 (3·2)	−10.17% (−16.17 to −4.17)	0.24 (0.09 to 0.62)	0.001
PPA	21/143 (14·7)	5/149 (3·35)	−11.33% (−17.81 to −4.84)	0.23 (0.09 to 0.62)	< 0.001
Within 12 months					
ITT	14/157 (8.9)	3/156 (1.9)	−6.99% (−11.95 to −2.04)	0.21 (0.06 to 0.73)	0.003
PPA	14/140 (10)	3/147 (2)	−7.95 (2.49 to 13.43)	5.92 (3.67 to 9.34)	0.002

Abbreviations: ITT, intention to treat analysis; PPA, per protocol analysis.

At the third month of follow‐up, the incidence of sarcopenia was 8.3% (26) in the total population, including five (3.2%) of the 156 patients in the bosentan group and 21 (13.4%) of the 157 patients in the placebo group [risk difference: −10.17% (−16.17 to −4.17), *p* = 0.001].

The cumulative incidence of sarcopenia within 6 months after randomization was 8.6% (*n* = 27) in the total population, including 5 (3.2%) of the 156 patients in the bosentan group and 22 (14%) of the 157 patients in the placebo group [risk difference: −10.81% (−16.9 to −4.71), *p* < 0.001]. During this time, one moderate sarcopenic patient was added to the control group.

At Month 9, the incidence was 8.3% (*n* = 26) in the total population, including five (3.2%) of the 156 patients in the bosentan group and 21 (13.4%) of the 157 patients in the placebo group [risk difference: −10.17% (−16.17 to −4.17), *p* = 0.001].

At the 12th‐month follow‐up, the incidence of sarcopenia was 5.4% (*n* = 17) in the total population, including three (1.9%) of the 156 patients in the bosentan group and 14 (8.9%) of the 157 patients in the placebo group [risk difference: −6.99% (−11.95 to −2.04), *p* = 0.003].

### 3.2 Subgroup Analysis

The association of bosentan with the risk of sarcopenia at 6 months after randomization was examined in subgroups based on age (< 50 years and ≥ 50 years), smoking status, hypertension, COPD, diabetes, BMI and cardiovascular disease (Table 3).

**TABLE 3:** The incidence rate of post‐COVID‐19 sarcopenia across subgroups at Month 6^a^.

	Sarcopenia				
	Placebo, *n*/*N* (%)	Bosentan, *n*/*N* (%)	Risk difference, bosentan vs placebo (95% CI)	Relative risk, bosentan vs placebo (95% CI)	*p*
Diabetes					
Yes	17/96 (17.7)	4/97(4. 1)	−13.58% (−22.18 to −4.98)	0.23 (0.08 to 0.67)	**0.002**
No	5/61 (8. 1)	0/59(0)	−8.19% (−15.08 to 1.31)	—	0.062
Hypertension					
Yes	14/67 (2.8)	3/48 (6·2)	−14.65% (−29.55 to ‐ 2.74)	0.29 (0.09 to 0.98)	**0.029**
No	8/90 (8.8)	2/108 (1.8)	−7.03% (−13.44 to 0.63)	0.20 (0.04 to 0.95)	0.052
COPD					
Yes	19/31 (61.2)	5/17 (29.4)	−31.88% (−59.50 to −4.25)	0.50 (0.21 to 1.03)	**0.041**
No	3/126 (2.3)	0/139 (0)	−2.32% (−4.92 to 0.27)	—	0.220
Smoke					
Yes	8/24 (33)	2/27 (7.4)	−25.93% (−47.21 to −4.63)	0.22 (0.05 to 0.94)	**0.046**
No	14/133 (8.8)	3/129 (2.3)	−8.20% (−14.03 to −2.37)	0.22 (0.06 to 0.75)	**0.007**
Age	317	96			
< 50	8/90 (8.9)	2/96 (0.20)	−6.80% (−13.34 to −0.26)	0.23 (0.05 to 1.07)	0.079
≥ 50	14/67 (20.8)	3/60 (0.5)	−15.9% (−27.08 to −4.70)	0.23 (0.07 to 0.79)	**0.008**
BMI					
< 25	13/77 (16.8)	3/80 (3.7)	−13.13 (−22.48 to −3.78)	0.22 (0.06 to 0.74)	**0.007**
≥ 25	9/80 (11.2)	2/76 (2.6)	−8.61 (−16.42 to −0.81)	0.23 (0.05 to 1.04)	**0.040**

*Note:* Bolded *p* values are statistically significant.

^a^ Intention to treat analysis.

In subgroup analysis based on smoking status, and BMI, the results were consistent with those in the overall population.

Bosentan significantly reduced risk of sarcopenia in subjects with diabetes, hypertension and age ≥ 50 years compared with the control group, but in subjects without diabetes, hypertension and age < 50 years, *p* values for the difference in risk between the bosentan and control groups were 0.062, 0.052 and 0.079, respectively.

Furthermore, in subgroup analysis based on COPD, bosentan was associated with reduced risk of sarcopenia only in subjects with COPD.

Regarding cardiovascular diseases, since sarcopenia was not observed in any of them, the analysis was not performed.

## 4 Discussion

The results of the present study showed that the early administration of bosentan tablets, 60 mg, twice daily for 1 month, reduced the absolute risk of sarcopenia in high‐risk COVID‐19 outpatients in the follow‐ups of 3, 6, 9 and 12 months after infection by 10.17%, 10.81%, 10.17% and 6.99%, respectively.

The incidence of sarcopenia was 8.3%, 8.6%, 8.3% and 5.4% at 3, 6, 9 and 12 months after randomization in the total population.

In this study, the incidence of sarcopenia was between 14% and 8.9% in the control group at different follow‐up times. Even though a small number of patients were hospitalized, the rate of sarcopenia seems to be high. This could be because high‐risk patients were included in this study. Nevertheless, studies have shown that prolonged COVID can occur with any severity of primary disease without the pathophysiology being clear [12].

We did not find any cohort studies reporting the incidence of sarcopenia in high‐risk COVID‐19 outpatients. In González et al.’s study, which followed 530 hospitalized patients with moderate to severe COVID‐19, the incidence of sarcopenia was 18.5% 3 months after infection [13]. In Levy et al.’s cohort study, sarcopenia was diagnosed in 16% and 4% of the 139 hospitalized COVID‐19 patients at 3 and 6 months after infection [14]. In the ‘Gemelli Against COVID‐19 Post‐Acute Care’ between April 2020 and February 2021, the prevalence of sarcopenia was reported as 19.5% in the 541 subjects recovered from COVID‐19 [15].

In the present study, bosentan reduced the incidence of sarcopenia significantly at all follow‐up time points compared to the control group. Bosentan is a drug that is an endothelin receptor antagonist and inhibits both ETA and ETB receptors. These receptors are mainly located in the endothelium and vascular smooth muscles. The stimulation of these receptors causes vasoconstriction. Sarcopenia is a disorder of all the skeletal muscles with gradual loss of function and mass and is generally age‐related but can also occur secondary to diseases at a younger age. In age‐related sarcopenia, ET‐1 has been shown to promote fibrosis and senescence in cultured myoblasts [3]. Also, a role has been shown for ET‐1 in the development of age‐related sarcopenia in mice [3].

There is increasing evidence on a link between endothelial dysfunction and sarcopenia. A study on 236 elderly women showed that there is a significant relationship between endothelial dysfunction and muscle strength [16]. In a cohort study of 77 patients with chronic kidney disease, sarcopenia was more common in patients with markers of atherosclerosis and endothelial dysfunction [17]. A systematic review has reported that endothelial dysfunction can be a predictor of sarcopenia [18]. In hyperglycaemic elderly patients, ET‐1 induces systemic inflammation and causes muscle myopathy by releasing cytokines such as IL‐6 [19].

In patients with COVID‐19, pro‐inflammatory cytokines (especially IL‐6) are increased. Long‐term exposure to IL‐6 causes muscle atrophy and wasting [20]. Bosentan also has anti‐inflammatory properties. Bosentan treatment has been shown to significantly reduce (ICAM‐1), IL‐6 and brain natriuretic peptide (BNP) in patients with pulmonary arterial hypertension [21].

There are no reports on the incidence of sarcopenia in clinical trials in which antiviral drugs or any drugs prescribed for COVID‐19 infection are used, but few clinical trials have followed the occurrence of several symptoms other than sarcopenia in long COVID, such as muscle pain and fatigue. These studies consider the reduction of virus load as the reason for the reduction of chronic COVID.

In a cohort study involving 282 000 US veterans, treatment with nirmatrelvir within 5 days of a positive SARS‐CoV‐2 test reduced the risk of post‐COVID‐19 condition, including cardiovascular disorders (dysrhythmia and ischemic heart disease), coagulation and hematologic disorders (pulmonary embolism and deep vein thrombosis), fatigue and malaise, acute kidney disease, muscle pain, neurologic disorders (neurocognitive impairment and dysautonomia) and shortness of breath, by 26% in those who had one risk factor for progression to severe COVID‐19, compared with the control group regardless of vaccination status [22].

In an observational cohort study among COVID‐19 outpatients, the risk of post–COVID‐19 symptoms reduced by 13% and 8% in those who received nirmatrelvir or molnupiravir, respectively [23].

In another study, the outpatient treatment of overweight and obese patients with metformin reduced the incidence of chronic COVID condition by about 41%, compared with the placebo [24].

In the present study, the subgroup analysis based on smoking status and BMI was consistent with results in the overall population. Regarding risk factors of diabetes, hypertension and age, *p* values were close to significant in subgroup analyses. Defined risk factors for COVID‐19 are conditions often associated with chronic vascular endothelial dysfunction due to increased production of endothelium‐derived contractile factors such as ET1. For this reason, these groups are at a high risk for COVID‐19 infection. The analysis of these subgroups strengthens our hypothesis that coronavirus damages body organs by destroying the endothelium. These findings might lead to a better understanding of who may benefit from bosentan therapy. However, subgroup analyses should be interpreted with caution, as this study was not designed to detect effects within subgroups. The wide range of the confidence interval also confirms this issue.

We previously reported that risk of hospitalization or death and thromboembolic events from any cause and duration of hospitalization were reduced significantly in the bosentan group compared to the placebo group during the 30‐day intervention. It seems that inhibition of endothelin receptors in the acute phase of infection may prevent acute and chronic complications of virus in in high‐risk outpatients.

### 4.1 Strength and Limitations

In our study, sarcopenia was evaluated at baseline, within 3 days of the onset of signs and symptoms of acute infection, and sarcopenic patients were excluded. The intake of calories, protein and vitamin D was also taken into account in the present study, and instructions were repeatedly given to the patients about this matter.

This study had some limitations. First, there is no consensus definition of acute sarcopenia, so the assessment and diagnosis of sarcopenia was made based on the AWGS, which is an age‐related sarcopenia. There are no cut‐off values for hand grip strength, gait speed and ASMI for all age groups, and the thresholds applied in this population are not based on a consensus definition. Second, the subgroup analyses should be interpreted with caution, as the study was not designed to detect the effects within the subgroups. Third, it would have been better to use *dual‐energy X‐ray absorptiometry* (DXA) instead of BIA to measure muscle mass, although AWGS 2019 recommends using either DXA or multifrequency BIA, both height‐adjusted, for measuring muscle mass in sarcopenia. Fourth, as we mentioned in our previously published article, it would have been better to continue the administration of bosentan for 2 months to observe the maximum effect, but we did not have permission from the ethics committee for this issue.

## 5 Conclusion

The present study shows that the risk of sarcopenia is high in high‐risk outpatients in chronic COVID. The inhibition of endothelin receptors in the acute phase of infection, which is involved in the pathophysiology of sarcopenia, may prevent the occurrence of sarcopenia.

## Conflicts of Interest

The authors declare no conflicts of interest.

## Supporting information


**Table S1** Cut‐off points for low handgrip strength (in kg) stratified by age and sex.

## Data Availability

Data will not be made available in a public repository as we have not obtained ethical clearance to share data publicly. However, on request from the corresponding author, data could be provided while maintaining anonymity, as stated in the protocol.
