# Supplementary material for: Early Administration of Bosentan in High‐Risk COVID‐19 Outpatients at Risk of Sarcopenia: A Randomized, Double‐Blind, Placebo‐Controlled Trial
Source: J Cachexia Sarcopenia Muscle. 2025 Mar 4;16(2):e13753. doi: 10.1002/jcsm.13753 (PMC11876846; doi:10.1002/jcsm.13753)
Supplement: Supplementary file 1 — Table S1 Cut‐off points for low handgrip strength (in kg) stratified by age and sex. [file JCSM-16-e13753-s001.docx]

| Supplementary Table 1. Cut-off points for low handgrip strength (in kg) stratified by age and sex | | | | |
| --- | --- | --- | --- | --- |
| Age (years) | 35- 40 | 41- 50 | 51- 60 | >60 |
| Women | 19 | 19 | 18 | 18 |
| Men | 34 | 32 | 29 | 28 |

**The cut-off points are the 25th percentile values ​​of the healthy South Asian adult population obtained from the Prospective Urban Rural Epidemiological Study (PURE).**
